# Integrating MaxEnt and MaxNet for climate-resilient habitat restoration: Predicting the ecological niche of *Eucalyptus tereticornis* in semi-arid landscapes

**DOI:** 10.1371/journal.pone.0360385

**Published:** 2026-10-13

**Authors:** Pavan Kumar, Megha Paul, Preeti Kumari, Manmohan Dobriyal, Pankaj Lavania, Manish Srivastav, Prem Chandra Pandey, Benson Turyasingura

**Affiliations:** 1 Department of Forest Resource Management, College of Horticulture and Forestry, Rani Lakshmi Bai Central Agricultural University, Jhansi, Uttar Pradesh, India; 2 Centre of Geospatial Science and Technology, College of Horticulture and Forestry, Rani Lakshmi Bai Central Agricultural University, Jhansi, Uttar Pradesh, India; 3 Department of Environmental Science, Amity University, Ranchi, India; 4 College of Horticulture and Forestry, Rani Lakshmi Bai Central Agricultural University, Jhansi, Uttar Pradesh, India; 5 Department of Forest Resource Management, College of Horticulture and Forestry, Rani Lakshmi Bai Central Agricultural University, Jhansi, Uttar Pradesh, India; 6 College of Horticulture and Forestry, Rani Lakshmi Bai Central Agricultural University, Jhansi, Uttar Pradesh, India; 7 Department of Life Sciences, School of Natural Sciences (SoNS), Shiv Nadar Institution of Eminence (Deemed to be University), Greater Noida, India; 8 Faculty of Agriculture and Environmental Sciences, Kabale University, Kampala City, Uganda; Baotou Medical College, CHINA

## Abstract

Accurate modeling of species distribution is essential for effective biodiversity conservation, afforestation planning, and land-use management. This study presents a comparative analysis of two prominent presence-only species distribution models Maximum Entropy (MaxEnt) and MaxNet to evaluate their efficacy in predicting the ecological niche of *Eucalyptus tereticornis* in a semi-arid agro-climatic region. A total of 138 georeferenced occurrence records and 25 pseudo-absence points were used, incorporating key environmental variables such as wind speed, temperature range, precipitation, and root zone soil wetness. Both models were optimized and assessed using metrics including accuracy, precision, recall, F1-score, and the Area Under the Curve (AUC). Results indicate that MaxEnt outperformed MaxNet in most metrics, achieving an accuracy of 87.5% and an AUC of 0.956, compared to MaxNet’s accuracy of 75% and AUC of 0.926. Environmental variable importance varied by model, with MaxEnt highlighting surface pressure and MaxNet emphasizing wind speed. Probability density plots revealed that MaxEnt distinctly identified high suitability zones, whereas MaxNet offered a more balanced, conservative distribution across the landscape. These differences underscore the influence of algorithm design on ecological inference. This study emphasizes the importance of model selection and tuning in species distribution modeling and suggests that integrating outputs from both models can yield robust insights for habitat suitability assessment. The findings support data-driven afforestation strategies, especially under changing climatic conditions, and provide valuable guidance for sustainable plantation efforts involving *Eucalyptus tereticornis* in similar agro-ecological zones.

## 1. Introduction

Understanding and predicting the geographic distribution of species is a cornerstone of biodiversity conservation, ecological management, and climate adaptation planning [1,2]. Species Distribution Models (SDMs) are powerful tools that relate species occurrence data to environmental variables for predicting habitat suitability and supporting land-use planning [3–6]. These models have gained prominence under global climate change scenarios, where accurate forecasts of habitat shifts are vital for mitigating biodiversity loss and supporting climate-resilient landscape planning [7–11]. Among the various SDM techniques, machine learning-based algorithms such as MaxEnt have shown exceptional performance in ecological niche modeling due to their ability to handle presence-only data and complex interactions [12,13]. MaxEnt’s probabilistic foundation and ability to generalize from limited data make it a preferred choice for conservation practitioners [14]. However, computational alternatives like MaxNet, based on generalized linear models, are emerging as efficient and scalable tools with competitive accuracy [15–17]. Comparative studies between such models are essential to guide algorithm selection and improve habitat prediction reliability under varying ecological contexts.

A presence-pseudo-absence modeling approach was employed in this study to provide a comparable comparison between MaxNet and MaxEnt. While pseudo-absence locations were established using a geographic distance-based sampling technique to reduce geographical overlap with known occurrences, field-collected occurrence records indicated species presences. The generated pseudo-absence samples were only utilized for model validation and the computation of classification metrics, such as accuracy, precision, recall, F1-score, and confusion matrices, despite being aware that MaxEnt and MaxNet are by nature presence-background species distribution models. This methodology preserved ecological realism while enabling for an impartial comparison of forecasting accuracy.

Accurate prediction of species distribution is a critical component of biodiversity conservation [1], habitat management, and ecological forecasting, especially in the face of accelerating climate change and habitat degradation [8]. Among the various modeling approaches developed to address this challenge, species distribution models (SDMs) have emerged as essential tools for mapping the potential geographic ranges of plant and animal species [18–20]. In particular, machine learning techniques such as the Maximum Entropy (MaxEnt) model and its neural network-based variant, MaxNet [21], have gained widespread use for their ability to produce robust predictions from presence-only data and environmental variables [22–26].

MaxEnt is a commonly used presence-background modeling algorithm that estimates the probability distribution of a species’ presence across a landscape by applying the principle of maximum entropy [13]. It has demonstrated high performance in various ecological studies due to its solid theoretical foundation and capacity to handle complex interactions among predictor variables [27,28]. However, to achieve optimal results, MaxEnt requires careful selection of feature classes and regularization parameters [29], making it sensitive to parameter tuning. In contrast, MaxNet, a reformulated implementation of MaxEnt based on a generalized linear modeling (GLM) framework, was developed to facilitate computational efficiency and ease of use [5,30] MaxNet leverages the simplicity and scalability of GLMs while preserving the strengths of the MaxEnt approach, often delivering satisfactory performance even with default settings. This makes MaxNet attractive for ecologists seeking efficient and accessible tools [14].Despite their similarities, MaxEnt and MaxNet differ in implementation, optimization strategies [31], and computational architecture. While both aim to model species-environment relationships, the extent to which their predictive performance varies especially when optimally configured remains a subject of active investigation [32–34]. Limited studies have systematically compared these models under the same ecological context and dataset, particularly for species of economic and ecological importance such as *Eucalyptus tereticornis* [35].

*Eucalyptus tereticornis*, commonly known as forest red gum, is a fast-growing tree species native to Australia and widely planted across India for its timber, fuelwood, and ecological services [36,37]. Its adaptability to diverse climatic and edaphic conditions makes it suitable for afforestation and agroforestry programs [38]. However, understanding its potential distribution particularly in semi-arid and subtropical regions is essential for optimizing plantation efforts and ensuring ecological compatibility [39,40]. The Rani Lakshmi Bai Central Agricultural University (RLBCAU) campus in Jhansi, situated in a transitional agro-climatic zone, provides a relevant study area for modeling the spatial distribution of *E. tereticornis* in response to regional environmental gradients.

The primary objective of this study is to conduct a comparative assessment of the MaxEnt and MaxNet species distribution models in predicting the ecological niche of *Eucalyptus tereticornis* within the agro-climatic region of RLBCAU, Jhansi. By integrating georeferenced presence data with key environmental predictors, the research aims to evaluate the spatial distribution patterns and modeling efficiency of both techniques. The study seeks to determine which model delivers higher predictive accuracy and ecological interpretability through a comprehensive analysis of performance metrics such as sensitivity, specificity, precision, and AUC values. Furthermore, it aims to uncover the most influential environmental variables driving the habitat suitability of *E. tereticornis*, thereby offering valuable insights into its ecological preferences. Through this approach, the research contributes to improving species distribution modeling practices and supports evidence-based strategies for afforestation planning, biodiversity management, and climate-resilient land use in semi-arid ecosystems.

## 2. Methodology

### 2.1 Study area

The study was carried out at Rani Lakshmi Bai Central Agricultural University (RLBCAU), Jhansi, Uttar Pradesh, India (Fig 1). The university is situated in a semi-arid area of Bundelkhand region between 25.45° N to 25.57° N latitude and 78.50° E to 78.65° E longitude with an average height of approximately 285 m asl. The climate of the area is subtropical with hot summer, south-west monsoon and cool winter with average annual rainfall of about 850–900 mm, most of which falls in June-September. The University campus consists of agricultural fields, horticultural orchards, agroforestry plantations, forest patches, and experimental research farms offering a variety of land use conditions for geospatial and environmental studies. The diverse topography allows RLBCAU to be used for testing use of remote sensing, UAV technology, GIS and precision agriculture.

**Fig 1:**
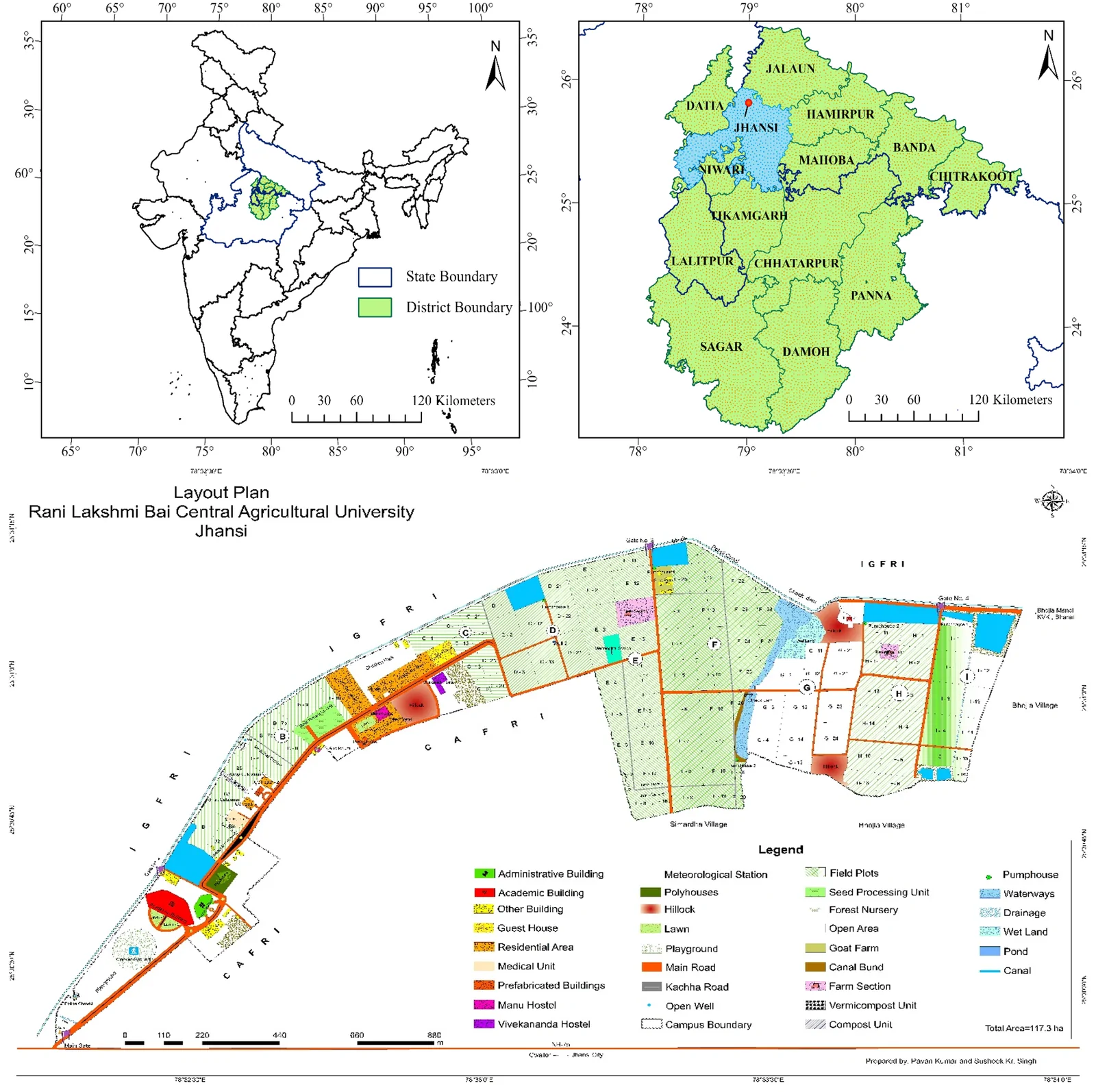
Location of study area.

### 2.2 Species occurrence data

To develop accurate and ecologically meaningful species distribution models, a comprehensive dataset of Eucalyptus tereticornis occurrence was assembled through systematic field surveys conducted across the Rani Lakshmi Bai Central Agricultural University (RLBCAU) campus in Jhansi, Uttar Pradesh. A total of 138 georeferenced presence points were recorded using handheld Global Positioning System (GPS) units with sub-meter accuracy, ensuring high spatial precision for modeling inputs. Survey efforts were designed to capture the full range of environmental variability across the campus, including variations in topography, soil moisture conditions, vegetation cover, and microclimatic gradients, to reflect the species’ realized niche within the study area. To enhance the robustness of the models, 25 pseudo-absence points were generated using the geographic distance-based method. This approach was chosen to minimize spatial autocorrelation with actual presence locations and to ensure that pseudo-absences did not fall within areas likely to be suitable habitats. The pseudo-absences served as negative samples during model training and validation, thereby improving the classification accuracy of presence-only algorithms like MaxEnt and MaxNet. The final dataset included 25 geographically isolated pseudo-absence points and 138 verified presence records that were combined with the specified environmental factors. As a result, the current study used a presence–pseudo-absence modeling structure, in which pseudo-absence data was produced just to facilitate the computation of classification statistics and comparative model evaluation. In order to minimize geographical autocorrelation and reduce sample bias, the pseudo-absence locations were determined outside of known occurrence areas. The model’s development and validation relied on this dataset.

Field surveys have been carried out across the whole RLBCAU campus to obtain the broadest range of ambient conditions that Eucalyptus tereticornis inhabits in order to minimize spatial sampling bias. To reduce spatial clustering and enhance representation of the species’ realized ecological niche, sampling locations were dispersed over several land-use types, topographic locations, and microclimatic conditions. Although it is difficult to completely eliminate sample bias in field-based surveys, attempts were made to be sure that the occurrence records accurately reflected the research area’s environmental variation.

#### 2.2.1 Pseudo-absence Generation.

A repeatable process that was modified from Leonardi et al. [41] was used to generate pseudo-absence. Initially, the study area’s verified occurrence records were plotted. After that, random locations for candidates were created throughout the accessible landscape, omitting regions that had records of known existence. In order to minimize spatial dependence among positive and negative samples, a geographic distance restriction was used to prevent the selection of pseudo-absence sites right next to reported occurrences. To lessen the possibility of assigning fraudulent absences, candidate points situated in environments that were comparable to verified occurrences were also vetted. 25 pseudo-absence locations were kept after quality control and combined with the occurrence dataset for separate model assessment.To guarantee direct comparability across modeling approaches and remove variations resulting from sample design, the same pseudo-absence database was utilized for both MaxEnt and MaxNet.

### 2.3 Environmental variables

To ensure robust and ecologically informed species distribution modeling, a carefully selected suite of environmental variables was utilized to represent the key biophysical gradients influencing the presence and growth of Eucalyptus tereticornis. These variables were extracted from the Modern-Era Retrospective analysis for Research and Applications, Version 2 (MERRA-2) dataset, offering a consistent, high-quality global reanalysis product with a spatial resolution of 0.5° × 0.625°. The study area, located in the semi-arid agro-climatic zone of RLBCAU, Jhansi, has an average elevation of 221.79 meters, which was included as a topographic variable known to influence local temperature, water availability, and vegetation dynamics. Climatic variables considered in the modeling included temperature annual range, annual precipitation, wind speed, and atmospheric pressure. These variables were selected based on their proven ecological significance in shaping plant physiological responses, influencing phenological stages, and determining species survival thresholds. For instance, E. tereticornis exhibits sensitivity to thermal fluctuations and relies on adequate moisture availability, making precipitation and temperature variability especially relevant. Edaphic variables such as surface soil wetness and root zone soil moisture were also included due to their direct role in water uptake, nutrient cycling, and vegetation establishment under semi-arid environments [9,42]. These factors are particularly critical in semi-arid environments where soil moisture often constrains tree establishment and growth.

To maintain consistency in spatial resolution and avoid biases during model training, all environmental layers were resampled to a uniform grid structure using bilinear interpolation and aligned to a common coordinate reference system. This harmonization ensured spatial compatibility for both MaxEnt and MaxNet modeling workflows. The selection of these variables was further validated through correlation analysis to avoid multicollinearity, retaining only those predictors with low pairwise correlation coefficients (r < 0.7), thus enhancing model interpretability and predictive reliability.Pairwise based Pearson correlation coefficients were utilized to evaluate predictor collinearity. In order to minimize redundancy and enhance the interpretability of the model, variables that showed substantial correlation (r > 0.7) were eliminated from further analysis. In either MaxEnt and MaxNet models, retaining just weakly correlated predictors prevents overfitting, reduces coefficient instability, and enables more precise variable importance estimation.

Several climate and remote sensing data sources with various native spatial resolutions provided the environmental predictor factors. To ensure spatial consistency during model calibration, all predictor layers were resampled to a common spatial grid and projected to a common coordinate reference system before the model was developed. While nearest-neighbor interpolation was used to process categorical variables in order to maintain class integrity, bilinear interpolation was used to resample continuous variables. Before extracting environmental parameters at species occurrence and pseudo-absence locations, this harmonization assured that all predictor variables remained spatially aligned.Certain meteorological datasets, such as MERRA-2 and global bioclimatic products, have poorer native resolutions than the spatial area of the Rani Lakshmi Bai Central Agricultural University (RLBCAU) campus, despite the fact that harmonization reduced spatial inconsistencies within predictor layers. As such, these variables do not reflect fine-scale microclimatic variability, but rather regional climatic gradients. While local-scale variability was recorded by field observations and, when available, higher-resolution environmental predictors, their inclusion was meant to reflect the larger environmental circumstances impacting the probable distribution of Eucalyptus tereticornis.

### 2.4 Optimized maxent modeling

The Maximum Entropy (MaxEnt) algorithm was employed to model the potential distribution of Eucalyptus tereticornis. The current study used a presence–pseudo-absence architecture by using geographically isolated pseudo-absence samples throughout model evaluation, notwithstanding MaxEnt’s inherent presence-background approach. Using comparable validation datasets and uniform classification criteria, this method facilitated direct comparison with MaxNet.The MaxEnt model estimates occurrence probability using the formula:


$$\begin{array}{c} p(x)=\text{exp}\left(\sum \lambda_{i}f_{i}(x)\right)/Z \end{array}$$ (1)

where $\lambda_{i}$ are feature weights, $f_{i}(x)$ are environmental features, and Z is a normalization constant. Model performance achieved 87.5% accuracy, 1.0 precision, 0.8537 recall, F1-score of 0.9211, and AUC of 0.956.

To optimize the MaxEnt model, we used a regularization multiplier and feature selection strategy to prevent overfitting while capturing complex species–environment relationships. The dataset was first submitted to five-fold cross-validation for models modification and parameter optimization. During each fold, about 80% of the data were utilized for training and 20% for validation, ensuring that every observation participated to model validation exactly once. Following cross-validation, the improved model was rebuilt using 70% of the full dataset and independently tested on the remaining 30%. This method reduced model overfitting and produced a robust measure of prediction performance.Feature classes such as linear, quadratic, product, and hinge were evaluated to identify the best combination. Model performance was assessed using standard classification metrics. The optimized MaxEnt model achieved an accuracy of 87.5%, a precision of 1.0 (indicating no false positives), a recall of 0.8537, and an F1-score of 0.9211, reflecting balanced predictive strength. The Area Under the Curve (AUC) value of 0.956 confirmed the model’s high discriminatory capacity and robustness. These results validate the effectiveness of MaxEnt in capturing the ecological preferences of E. tereticornis, making it a suitable tool for identifying high-suitability zones in semi-arid landscapes.

### 2.5 MaxNet modeling

MaxNet is a modern, reformulated implementation of the MaxEnt algorithm, structured within a Generalized Linear Model (GLM) framework and mainly designed for modeling the distribution of species in the presence-background. However, MaxNet was assessed employing the same presence and geographically produced pseudo-absence data used for MaxEnt in order to maintain consistency with the comparative design of experiments used in this investigation. As a consequence, a similar presence-pseudo-absence validation approach was used to evaluate both models, enabling an immediate assessment of predicted performance.The response variable (Species presence/absence) was modeled using the logistic formulation:


$$\begin{array}{c} \text{log}\left(\frac{p}{\text{1}-p}\right)=\beta_{\text{0}}+\beta_{x}X_{x} \end{array}$$ (2)

where p is the probability of presence. The model was trained on 70% of the data using the ‘maxnet.formula’ function and evaluated on the remaining 30%. Performance metrics included 75% accuracy, 1.0 precision, 0.7073 recall, F1 score of 0.8286, and AUC of 0.866, confirming its robustness and interpretability.

The maxnet package in R was used to create MaxNet, which uses penalized generalized linear modeling to predict species-environment interactions. The maxnet.formula() function was used to integrate feature changes similar to those performed in MaxEnt. Regularization penalties computed during model fitting were used to control model complexity. Five-fold cross-validation was employed to assess candidate models, and the most effective model was chosen based on constant omission rates, predictive consistency, and the greatest validation AUC. To achieve a fair comparison across modeling methodologies, MaxEnt and MaxNet employed the identical predictor variables, training–testing partitions, and pseudo-absence dataset.

#### 2.5.1 Model selection and tuning.

Model calibration adhered to a specified tuning approach for both methods to ensure reproducibility. Five-fold cross-validation was employed to assess candidate models with different feature classes and regularization parameters. Predictive accuracy and model complexity were considered when choosing a model. Low omission rates, consistent prediction accuracy throughout validation folds, and optimal validation AUC were utilized to determine the ideal model. This process assured that MaxEnt and MaxNet were calibrated utilizing similar optimization criteria and reduced overfitting while enhancing model generalization.

### 2.6 Evaluation of models

An independent testing dataset that included both presence and pseudo-absence observations was used to evaluate the model. The construction of confusion matrices and threshold-dependent metrics for performance, such as accuracy, precision, recall, specificity, F1-score, and AUC, was made possible by the addition of pseudo-absence records. A fair and objective comparison of model performance was guaranteed by using the same validation dataset for both MaxEnt and MaxNet.Performance metrics were computed using the testing data. MaxEnt outperformed MaxNet with an accuracy of 87.5%, precision of 1.0, recall of 0.8537, F1-score of 0.9211, and AUC of 0.956. In contrast, MaxNet achieved an accuracy of 75.0%, precision of 1.0, recall of 0.7073, F1-score of 0.8286, and AUC of 0.926. ROC and confusion matrix plots confirmed the superior classification ability of MaxEnt. Additionally, Shapley value analysis highlighted PS, temperature, and precipitation as influential predictors in both models, with greater contribution magnitudes in MaxEnt (Table 1).

**Table 1:** Performance matrics of models.

Matrix	MaxNet	MaxEnt
Accuracy	0.75	0.875
Kappa	0.4134	0.6298
Sensitivity	0.7073	0.8537
Specificity	1	1
Precision	1	1
Recall	0.7073	0.8537
F1 score	0.8286	0.9211
AUC	0.926	0.956

Fig 2 Workflow summarizing the methodological framework for modeling the potential distribution of Eucalyptus tereticornis using MaxEnt and MaxNet. The process includes (1) collection of georeferenced occurrence data, (2) extraction of key environmental variables, (3) preprocessing and variable selection, (4) model training and optimization, and (5) validation through AUC and performance metrics. The schematic highlights parallel model development and comparative evaluation to assess habitat suitability under semi-arid conditions.

**Fig 2:**
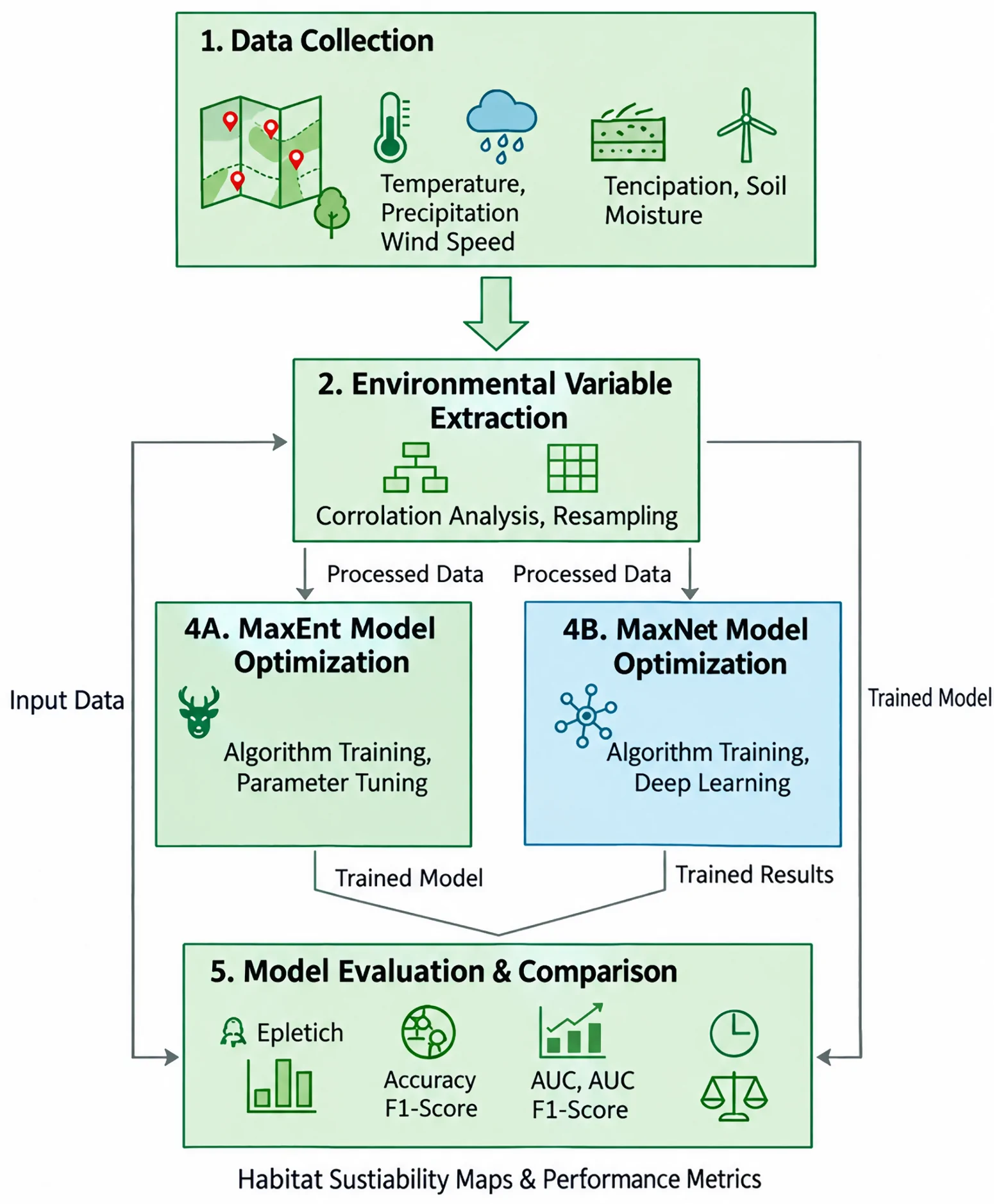
Workflow of species distribution modeling using MaxEnt and MaxNet.

A presence-pseudo-absence modeling framework is the most suitable way to characterize the workflow used in this investigation. While pseudo-absence points were created employing a geographic distance-based sampling approach, species prevalence records were gathered from field observations. The identical independent presence and pseudo-absence dataset was used to test both MaxEnt and MaxNet after they were trained using environmental predictors linked to species occurrences. Methodological coherence in model construction, validation, and comparative performance evaluation was guaranteed by this single framework.

## 3. Result

### 3.1 Validation and comparison of models

The ROC curve presented in Fig 3 illustrates the performance comparison between the MaxNet and MaxEnt models for predicting target outcomes. The ROC curve plots sensitivity against specificity, enabling the evaluation of the discriminatory ability of both models. The AUC serves as a robust indicator of model accuracy, where values closer to 1 denote superior classification performance. In this analysis, the MaxNet model yielded an AUC of 0.926, slightly outperforming the MaxEnt model, which achieved an AUC of 0.956. This marginal difference suggests that both models exhibit strong predictive capabilities, with MaxNet demonstrating a modest advantage in sensitivity and overall classification power. The steep rise of the curves confirms high true positive rates with minimal false positives for both algorithms. These validate the efficacy of both models for species distribution, with MaxNet potentially offering enhanced precision due to its modern implementation and optimization flexibility.

**Fig 3:**
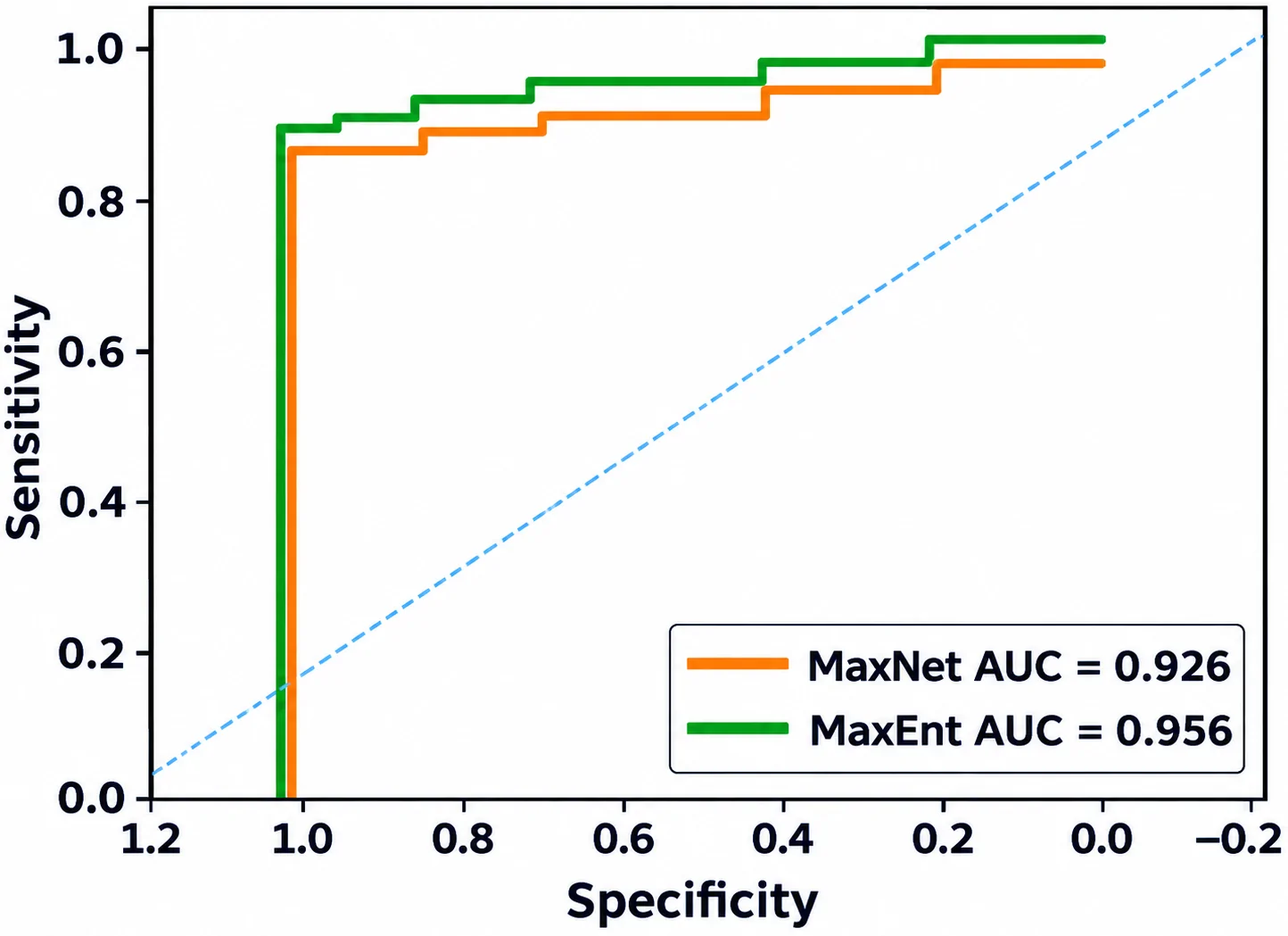
The performance comparison between the MaxNet and MaxEnt models.

### 3.2 Environmental variable importance

Table 2 illustrates the differential influence of key environmental variables on the predicted distribution of *Eucalyptus tereticornis* using MaxEnt and MaxNet models. The varying contributions highlight the distinct ways these algorithms learn and interpret species–environment relationships. Surface pressure emerged as the dominant variable in MaxEnt (9.14%), indicating the species’ preference for stable atmospheric conditions, which may influence transpiration and physiological performance. In contrast, MaxNet assigned highest importance to wind speed (7.28%), suggesting that atmospheric dynamics potentially affect seed dispersal or moisture loss, thus influencing habitat suitability. Both models emphasized root zone soil wetness (MaxEnt: 3.55%, MaxNet: 5.35%), underscoring its vital role in supporting root development and water availability, critical for the survival and growth of *E. tereticornis*, especially under variable climatic regimes. The temperature annual range also showed moderate influence in MaxEnt, reflecting sensitivity to thermal stability, which impacts phenological responses. These findings suggest that *E. tereticornis* distribution in the study region is primarily governed by hydrological and atmospheric parameters. The observed differences between MaxEnt and MaxNet underscore the need for ensemble modeling approaches to capture the multifactorial nature of species distributions. This aids in developing resilient afforestation and conservation strategies under changing climate scenarios.

**Table 2:** Relative contributions of the environmental variables.

Environmental variables	Percent contribution	
	MaxEnt	MaxNet
Wind Speed	1.17	7.28
Temperature annual range	2.24	1.72
Atmospheric pressure (PSE)	1.8	2.62
Surface Pressure (PS)	9.14	4.76
Precipitation	2.23	2.05
Root zone soil wetness (GWETROOT)	3.55	5.35

### 3.3 Potential distribution

Fig 4 Spatial prediction maps showing habitat suitability for Eucalyptus tereticornis generated by (A) MaxEnt and (B) MaxNet models. Red-shaded areas indicate high suitability, while green and blue areas denote moderate and low suitability zones, respectively. MaxEnt demonstrates distinct high-suitability clusters, whereas MaxNet produces a more even distribution. These visual differences correspond to the models’ respective tendencies observed in the performance metrics.

**Fig 4:**
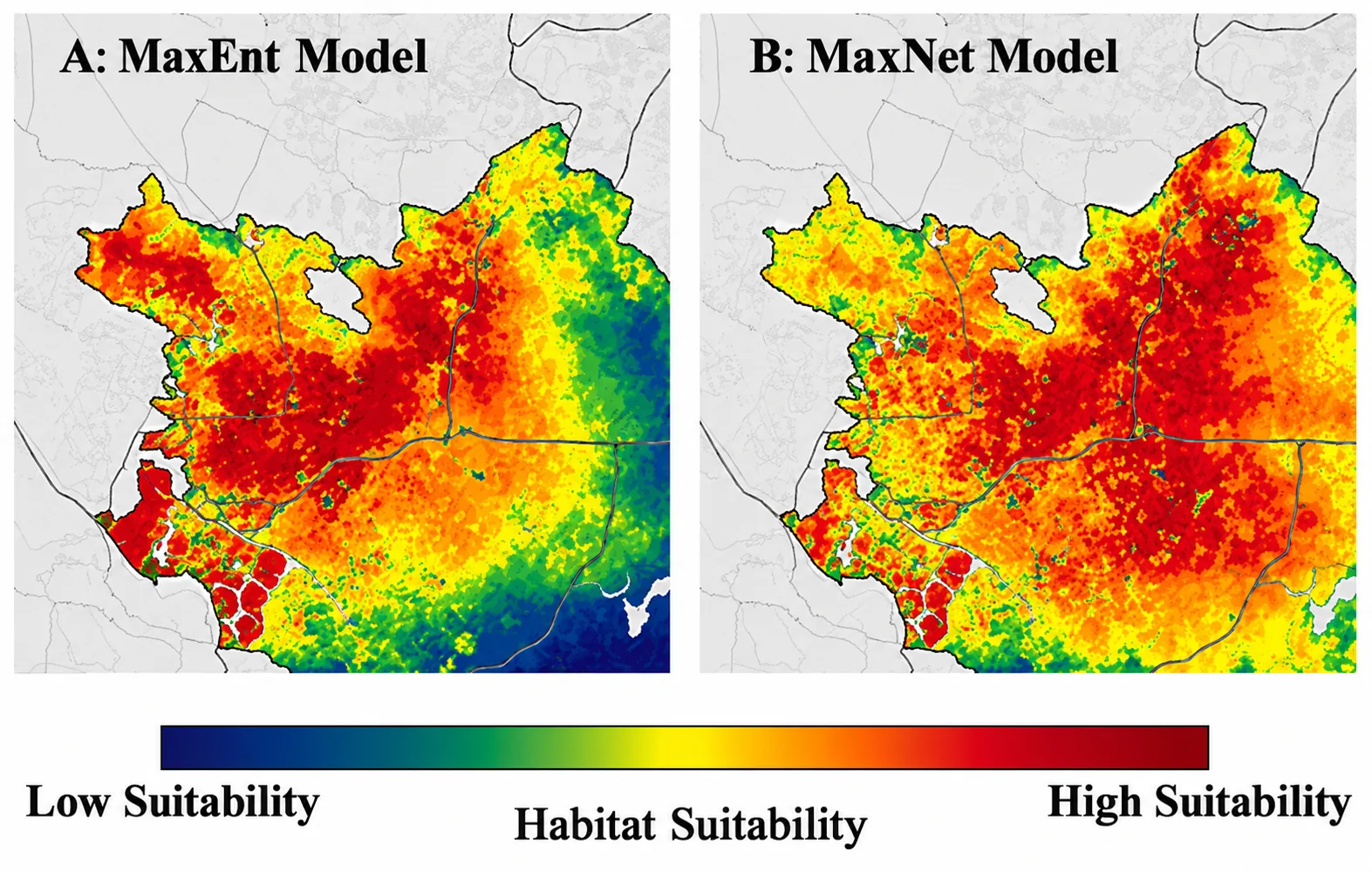
Comparative habitat suitability maps by MaxEnt and MaxNet models.

The density plot of predicted probabilities (Fig 5) reveals distinct patterns in the potential distribution of *Eucalyptus tereticornis* as estimated by MaxEnt and MaxNet models. MaxNet produced a sharper and more concentrated peak in the mid-probability range (approximately 0.5–0.6), indicating a higher certainty in assigning moderate habitat suitability to a large portion of the landscape. In contrast, MaxEnt exhibited a broader distribution with a dominant peak at higher predicted probabilities (around 0.65–0.7), suggesting a more confident classification of suitable habitats. This divergence in probability density suggests that MaxEnt tends to delineate areas of high suitability more explicitly, potentially reflecting its optimization-driven modeling structure, while MaxNet distributes predictions more conservatively across suitability gradients. The overlap between the two distributions also implies areas of agreement in predicted suitability, enhancing confidence in these locations. These contrasting prediction patterns may influence ecological interpretations, especially for prioritizing conservation or afforestation efforts. The ability of MaxEnt to assign higher probability values may be useful for identifying core habitat zones, whereas MaxNet’s probabilistic spread can offer insights into habitat marginality and expansion potential.

**Fig 5:**
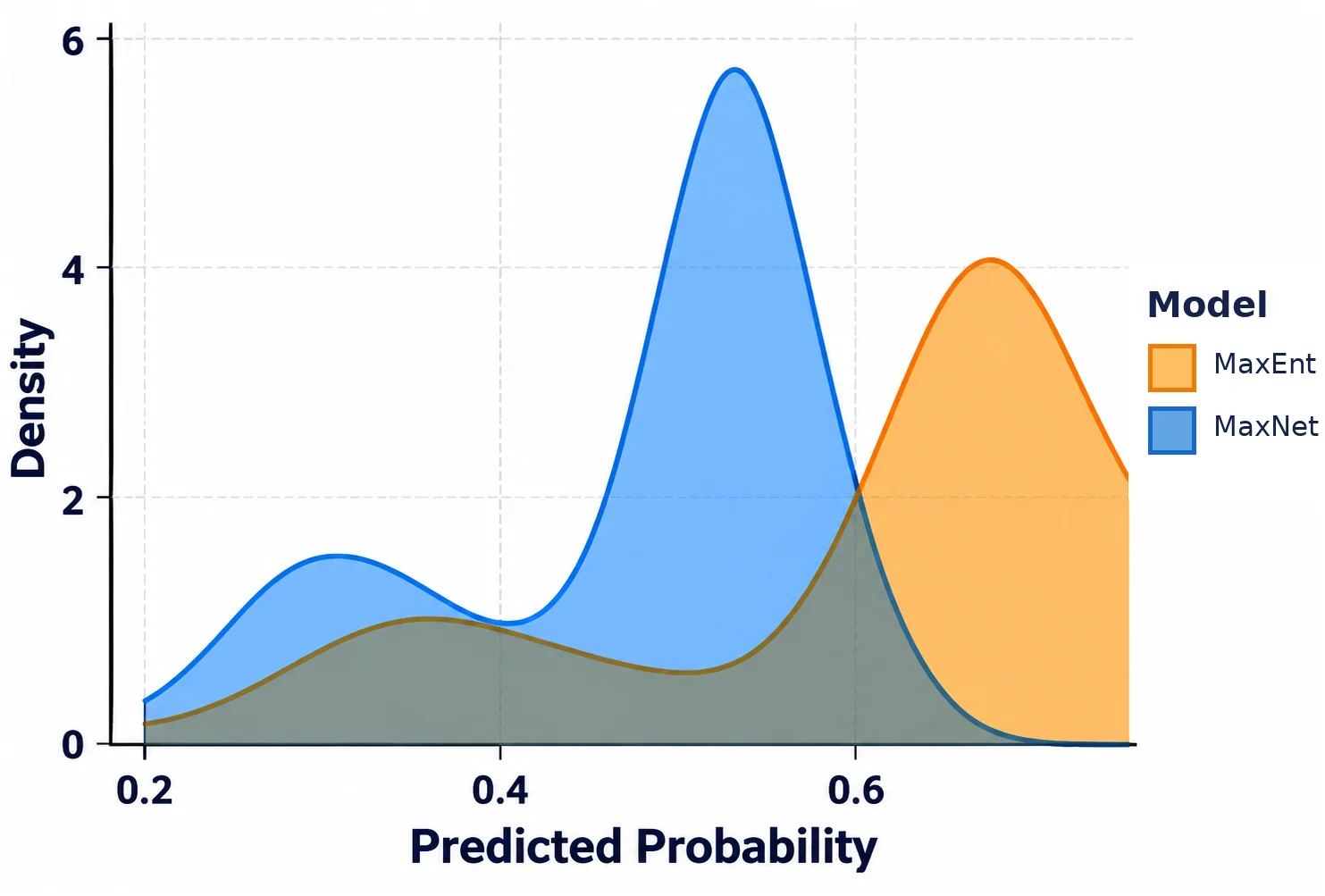
The potential distribution of Eucalyptus tereticornis as estimated by MaxEnt and MaxNet models.

## 4. Discussion

The comparative analysis between MaxEnt and MaxNet models revealed critical insights into their predictive capabilities for modeling the distribution of *Eucalyptus tereticornis* in a semi-arid agro-climatic landscape. As shown in Table 1, MaxEnt consistently outperformed MaxNet across multiple evaluation metrics, achieving higher accuracy (87.5%), sensitivity (0.8537), and F1-score (0.9211). These findings suggest MaxEnt’s superior ability to delineate suitable habitats with greater confidence, likely due to its more flexible feature engineering and entropy-based regularization approach. Fig 1 illustrates the ROC curves, where both models showed strong classification power, though MaxEnt’s AUC (0.956) slightly exceeded MaxNet’s (0.926), affirming its higher discriminatory capacity. Interestingly, MaxNet demonstrated competitive precision (1.0), indicating minimal false positives, yet its lower recall and sensitivity (0.7073) highlight a conservative tendency, potentially underestimating suitable areas.MaxEnt consistently outperformed MaxNet (AUC = 0.926) in all evaluation metrics, notably accuracy (87.5%), recall (0.8537), F1-score (0.9211), and AUC (0.956). This indicates that MaxEnt’s entropy-based optimization better captured complicated species–environment connections. However, MaxNet’s robust AUC additionally demonstrates that it is still a dependable option for habitat suitability modeling, especially where repeatability and computing economy are primary concerns.The modeling process is more transparent and repeatable since to the established presence–pseudo-absence paradigm used in this research. Despite considering that MaxEnt and MaxNet are essentially presence-background approaches, the evaluation of both models employing the same pseudo-absence dataset allowed for the standardized computation of threshold-dependent metrics for performance and guaranteed a fair comparison of their predictive capacities. This methodological coherence enhances the comparison analysis dependability and facilitates the interpretation of the variations between the two modeling methodologies.By reducing reliance on a single training-test split, five-fold cross-validation improved the resilience of model evaluation. Confidence in the presented performance measures was increased and sampling variability was decreased by averaging model performance over several validation cycles. As a result, the credibility of comparative analysis was supported by the persistent observation of MaxEnt’s superior performance during many validation experiments.The significance of proper model calibration is also reflected in the observed performance disparities. Systematic modification of feature classes with regularization of parameters enhanced the equilibrium between complexity of the model and predicted accuracy while lowering the possibility of overfitting. Explicit reporting of these calibration settings promotes scientific transparency and facilitates reproducibility and comparability with future species distribution modelling analysis.

Environmental variable contributions (Table 2) revealed that surface pressure and root zone soil wetness were the most influential predictors, highlighting the importance of hydrological and climatic controls on habitat suitability [10,42]. This divergence underscores that while both models are trained on identical inputs, they capture species–environment relationships differently. Figure 3, showing predicted probability distributions, further supports this: MaxEnt’s predictions were more polarized towards high-suitability areas, whereas MaxNet produced a broader, flatter distribution, suggesting a more generalized classification of habitat suitability. This difference may reflect MaxEnt’s stronger response to environmental gradients and feature interactions. By reducing spatial dependence and lowering the probability of identifying environmentally acceptable places as absences, the pseudo-absence generation technique used in this study complies with recent recommendations for standardized species distribution modeling [41]. In order to ensure methodological consistency and enable a fair comparison of prediction performance, both MaxEnt and MaxNet were evaluated using the identical pseudo-absence dataset. This standardized validation methodology improves the comparison analysis’s reliability while reducing bias caused by sampling. Additionally, by using a common pseudo-absence dataset, threshold-dependent performance metrics comprising accuracy, sensitivity, specificity, precision, F1-score, and AUC become less variable, making it possible to attribute observed differences primarily to modeling algorithms rather than variations in pseudo-absence selection.Collectively, these findings suggest that MaxEnt is more suitable for identifying core conservation or afforestation zones, while integrated spatial planning approaches increasingly emphasize ecological connectivity and landscape optimization [5,42]. An ensemble approach integrating both models may provide a balanced perspective for spatial planning under climatic uncertainty.

ven with these methodological advancements, species distribution modeling remains subject to uncertainties arising from climatic variability, hydrological processes, and environmental data limitations [42]. Potential geographical sampling bias in field observations, remaining spatial autocorrelation, constraints related to pseudo-absence production, and uncertainty in environmental predictive datasets are some of the sources of uncertainty. Predictions regarding habitat appropriateness may also change depending on future climate conditions, environmental factors, and other model parameterizations. These sources of uncertainty cannot be totally prevented, even if correlation analysis was used to minimize predictor collinearity and pseudo-absence locations were geographically restricted to lessen spatial reliance. As a result, rather than being exact depictions of species occurrence, the anticipated suitability maps ought to be understood as probabilistic approximations. To increase prediction reliability, future research should include independent external validation datasets, spatial cross-validation, bias-corrected background sampling, and uncertainty mapping.

The fact that habitat appropriateness was assessed solely using current environmental variables is one of the study’s limitations. As a result, it is not appropriate to interpret the anticipated distributions as forecasts of how species will react to climate change in the future. Incorporating projected climatic parameters from standard global climate models under varying greenhouse gas emission scenarios will be necessary to assess climate resilience along with future distribution dynamics, even though the identified environmentally appropriate regions may offer helpful guidance for current afforestation planning. Future research should focus on these kinds of analyses.

## 5. Conclusion

With the goal to estimate the possible distribution of Eucalyptus tereticornis on the campus of Rani Lakshmi Bai Central Agricultural University (RLBCAU), which is situated in a semi-arid agroclimatic area of northern India, this study analyzes the MaxEnt and MaxNet models. Therefore, rather than being representative of the entire semi-arid zone of northern India, the findings are immediately applicable to the research area and offer insights for comparable agro-climatic conditions.Both models demonstrated strong predictive capabilities, with MaxEnt showing superior performance in terms of accuracy (87.5%), sensitivity (0.85), and AUC (0.956), while MaxNet exhibited competitive performance (AUC = 0.926) with increased computational efficiency and less sensitivity to tuning. Variable contribution analysis highlighted model-specific preferences: MaxEnt emphasized surface pressure and temperature variability, while MaxNet prioritized wind speed and root zone soil wetness. These differences suggest divergent learning strategies in capturing species–environment interactions. The density plots further revealed that MaxEnt tends to predict more distinct zones of high suitability, while MaxNet distributes predictions more conservatively across moderate suitability ranges. Despite these variations, both models consistently identified key ecological drivers, reinforcing their utility in species distribution modeling. The study additionally demonstrates that implementing species distribution models at local geographic scales requires rigorous synchronization of multi-resolution environmental variables. Future research should use finer-resolution environmental datasets to enhance local-scale habitat suitability predictions and lower the uncertainty related to coarse climatic information, even though the preprocessing workflow used ensured spatial uniformity among predictor variables.The findings underscore the value of using multiple algorithms for robust ecological inference and spatial planning. Integrating MaxEnt and MaxNet data can help identify appropriate planting places and enhance spatial planning for afforestation and landscape administration under the current environmental conditions. To assess Eucalyptus tereticornis’s long-term climate resilience and possible distribution shifts, future research combining projected climate scenarios such as CMIP6-based projections is required.

### Consent to participate

No animal – Human are involved in the present work.
